# Machine learning can predict survival of patients with heart failure from serum creatinine and ejection fraction alone

**DOI:** 10.1186/s12911-020-1023-5

**Published:** 2020-02-03

**Authors:** Davide Chicco, Giuseppe Jurman

**Affiliations:** 1 Krembil Research Institute, Toronto, Ontario, Canada; 2 Fondazione Bruno Kessler, Trento, Italy

**Keywords:** Cardiovascular heart diseases, Heart failure, Serum creatinine, Ejection fraction, Medical records, Feature ranking, Feature selection, Biostatistics, Machine learning, Data mining, Biomedical informatics

## Abstract

**Background:**

Cardiovascular diseases kill approximately 17 million people globally every year, and they mainly exhibit as myocardial infarctions and heart failures. Heart failure (HF) occurs when the heart cannot pump enough blood to meet the needs of the body.Available electronic medical records of patients quantify symptoms, body features, and clinical laboratory test values, which can be used to perform biostatistics analysis aimed at highlighting patterns and correlations otherwise undetectable by medical doctors. Machine learning, in particular, can predict patients’ survival from their data and can individuate the most important features among those included in their medical records.

**Methods:**

In this paper, we analyze a dataset of 299 patients with heart failure collected in 2015. We apply several machine learning classifiers to both predict the patients survival, and rank the features corresponding to the most important risk factors. We also perform an alternative feature ranking analysis by employing traditional biostatistics tests, and compare these results with those provided by the machine learning algorithms. Since both feature ranking approaches clearly identify serum creatinine and ejection fraction as the two most relevant features, we then build the machine learning survival prediction models on these two factors alone.

**Results:**

Our results of these two-feature models show not only that serum creatinine and ejection fraction are sufficient to predict survival of heart failure patients from medical records, but also that using these two features alone can lead to more accurate predictions than using the original dataset features in its entirety. We also carry out an analysis including the follow-up month of each patient: even in this case, serum creatinine and ejection fraction are the most predictive clinical features of the dataset, and are sufficient to predict patients’ survival.

**Conclusions:**

This discovery has the potential to impact on clinical practice, becoming a new supporting tool for physicians when predicting if a heart failure patient will survive or not. Indeed, medical doctors aiming at understanding if a patient will survive after heart failure may focus mainly on serum creatinine and ejection fraction.

## Background

Cardiovascular diseases (CVDs) are disorders of the heart and blood vessels including, coronary heart disease (heart attacks), cerebrovascular diseases (strokes), heart failure (HF), and other types of pathology [1]. Altogether, cardiovascular diseases cause the death of approximately 17 million people worldwide annually, with fatalities figures on the rise for first time in 50 years the United Kingdom [2]. In particular, heart failure occurs when the heart is unable to pump enough blood to the body, and it is usually caused by diabetes, high blood pressure, or other heart conditions or diseases [3].

The clinical community groups heart failure into two types based on the ejection fraction value, that is the proportion of blood pumped out of the heart during a single contraction, given as a percentage with physiological values ranging between 50% and 75%. The former is heart failure due to reduced ejection fraction (HFrEF), previously known as *heart failure due to left ventricular (LV) systolic dysfunction* or *systolic heart failure* and characterized by an ejection fraction smaller than 40% [4]. The latter is heart failure with preserved ejection fraction (HFpEF), formerly called *diastolic heart failure* or *heart failure with normal ejection fraction*. In this case, the left ventricle contracts normally during systole, but the ventricle is stiff and fails to relax normally during diastole, thus impairing filling [5–10].

For the quantitative evaluation of the disease progression, clinicians rely on the New York Heart Association (NYHA) functional classification, including four classes ranging from no symptoms from ordinary activities (Class I) to a stage where any physical activity brings on discomfort and symptoms occur at rest (Class IV). Despite its widespread use, there is no consistent method of assessing the NYHA score, and this classification fails to reliably predict basic features, such as walking distance or exercise tolerance on formal testing [11].

Given the importance of a vital organ such as the heart, predicting heart failure has become a priority for medical doctors and physicians, but to date forecasting heart failure-related events in clinical practice usually has failed to reach high accuracy [12].

In this context, electronic health records (EHRs, also called *medical records*) can be considered a useful resource of information to unveil hidden and non-obvious correlations and relationships between patients’ data, not only for research but also for clinical practice [13, 14] and for debunking traditional myths on risk factors [15, 16]. To this aim, several screening studies have been conducted in the last years, covering different conditions and demographics and with different data sources, to deepen the knowledge on the risk factors. Among them, it is worth mentioning the PLIC study [17], where EHRs, blood test, single-nucleotide polymorphisms (SNPs), carotid ultrasound imaging, and metagenomics data have been collected in a four-visit longitudinal screening throughout 15 years in Milan (Italy, EU) to support a better assessment of cardiovascular disease risk.

Machine learning applied to medical records, in particular, can be an effective tool both to predict the survival of each patient having heart failure symptoms [18, 19], and to detect the most important clinical features (or risk factors) that may lead to heart failure [20, 21]. Scientists can take advantage of machine learning not only for clinical prediction [22, 23], but also for feature ranking [24]. Computational intelligence, especially, shows its predictive power when applied to medical records [25, 26], or coupled with imaging [27–29]. Further, deep learning and meta-analysis studies applied to this field have also recently appeared in the literature [30–33], improving on human specialists’ performance [34], albeit showing lower accuracy (0.75 versus 0.59).

Modeling survival for heart failure (and CVDs in general) is still a problem nowadays, both in terms of achieving high prediction accuracy and identifying the driving factors. Most of the models developed for this purpose reach only modest accuracy [35], with limited interpretability from the predicting variables [36]. More recent models show improvements, especially if the survival outcome is coupled with additional targets (for example, hospitalization [37]). Although scientists have identified a broad set of predictors and indicators, there is no shared consensus on their relative impact on survival prediction [38]. As pointed out by Sakamoto and colleagues [39], this situation is largely due to a lack of reproducibility, which prevents drawing definitive conclusions about the importance of the detected factors. Further, this lack of reproducibility strongly affects model performances: generalization to external validation datasets is often inconsistent and achieves only modest discrimination. Consequently, risk scores distilled from the models suffer similar problems, limiting their reliability [40]. Such uncertainty has led to the proliferation of new risk scores appearing in the literature in the last years, with mixed results [41–47]. As a partial solution to improve models’ effectiveness, recent published studies included cohorts restricted to specific classes of patients (for example, elderly or diabetic) [48, 49]. These attempts have led to tailored models and risk scores [50, 51] with better but still not optimal performance.

In this paper, we analyze a dataset of medical records of patients having heart failure released by Ahmad and colleagues [52] in July 2017. Ahmad and colleagues [52] employed traditional biostatistics time-dependent models (such as Cox regression [53] and Kaplan–Meier survival plots [54]) to predict mortality and identify the key features of 299 Pakistan patients having heart failure, from their medical records. Together with their analysis description and results, Ahmad and coworkers made their dataset publicly available online (“Dataset” section), making it freely accessible to the scientific community [55]. Afterwards, Zahid and colleagues [56] analyzed the same dataset to elaborate two different sex-based mortality prediction models: one for men and one for women. Although the two aforementioned studies [52, 56] presented interesting results, they tackled the problem by standard biostatistics methods, leaving room for machine learning approaches. We aim here to fill this gap by using several data mining techniques first to predict survival of the patients, and then to rank the most important features included in the medical records. As major result, we show that the top predictive performances can be reached by machine learning methods with just two features, none of them coming unexpected: one is ejection fraction, and the other is serum creatinine, well known in the literature as a major driver of heart failure [57–62], and also a key biomarker in renal dysfunction [63–65].

In particular, we first describe the analyzed dataset and its features (“Dataset” section), and then the methods we employed for survival prediction and feature ranking (“Methods” section). In the Results section (“Results” section), we report the survival prediction performances obtained through all the employed classifiers (“Survival machine learning prediction on all clinical features” section), the ranking of the features obtained through traditional biostatistics techniques and machine learning (“Feature ranking results” section), and the survival prediction performances achieved by employing only the top two features identified through feature ranking (ejection fraction and serum creatinine, “Survival machine learning prediction on serum creatinine and ejection fraction alone” section). Later, we report and describe the results of the analysis that includes the patients’ follow-up time (Table 11). Finally, we discuss the results (“Discussion” section) and draw some conclusions at the end of the manuscript (“Conclusions” section).

## Dataset

We analyzed a dataset containing the medical records of 299 heart failure patients collected at the Faisalabad Institute of Cardiology and at the Allied Hospital in Faisalabad (Punjab, Pakistan), during April–December 2015 [52, 66]. The patients consisted of 105 women and 194 men, and their ages range between 40 and 95 years old (Table 1). All 299 patients had left ventricular systolic dysfunction and had previous heart failures that put them in classes III or IV of New York Heart Association (NYHA) classification of the stages of heart failure [67].

**Table 1:** Meanings, measurement units, and intervals of each feature of the dataset

Feature	Explanation	Measurement	Range
Age	Age of the patient	Years	[40,..., 95]
Anaemia	Decrease of red blood cells or hemoglobin	Boolean	0, 1
High blood pressure	If a patient has hypertension	Boolean	0, 1
Creatinine phosphokinase	Level of the CPK enzyme in the blood	mcg/L	[23,..., 7861]
(CPK)			
Diabetes	If the patient has diabetes	Boolean	0, 1
Ejection fraction	Percentage of blood leaving	Percentage	[14,..., 80]
	the heart at each contraction		
Sex	Woman or man	Binary	0, 1
Platelets	Platelets in the blood	kiloplatelets/mL	[25.01,..., 850.00]
Serum creatinine	Level of creatinine in the blood	mg/dL	[0.50,..., 9.40]
Serum sodium	Level of sodium in the blood	mEq/L	[114,..., 148]
Smoking	If the patient smokes	Boolean	0, 1
Time	Follow-up period	Days	[4,...,285]
(target) death event	If the patient died during the follow-up period	Boolean	0, 1

mcg/L: micrograms per liter. mL: microliter. mEq/L: milliequivalents per litre

The dataset contains 13 features, which report clinical, body, and lifestyle information (Table 1), that we briefly describe here. Some features are binary: anaemia, high blood pressure, diabetes, sex, and smoking (Table 1). The hospital physician considered a patient having anaemia if haematocrit levels were lower than 36% [52]. Unfortunately, the original dataset manuscript provides no definition of high blood pressure [52].

Regarding the features, the creatinine phosphokinase (CPK) states the level of the CPK enzyme in blood. When a muscle tissue gets damaged, CPK flows into the blood. Therefore, high levels of CPK in the blood of a patient might indicate a heart failure or injury [68]. The ejection fraction states the percentage of how much blood the left ventricle pumps out with each contraction. The serum creatinine is a waste product generated by creatine, when a muscle breaks down. Especially, doctors focus on serum creatinine in blood to check kidney function. If a patient has high levels of serum creatinine, it may indicate renal dysfunction [69]. Sodium is a mineral that serves for the correct functioning of muscles and nerves. The serum sodium test is a routine blood exam that indicates if a patient has normal levels of sodium in the blood. An abnormally low level of sodium in the blood might be caused by heart failure [70]. The death event feature, that we use as the target in our binary classification study, states if the patient died or survived before the end of the follow-up period, that was 130 days on average [52]. The original dataset article [52] unfortunately does not indicate if any patient had primary kidney disease, and provides no additional information about what type of follow-up was carried out. Regarding the dataset imbalance, the survived patients (death event = 0) are 203, while the dead patients (death event = 1) are 96. In statistical terms, there are 32.11% positives and 67.89% negatives.

As done by the original data curators [52], we represented this dataset as a table having 299 rows (patients) and 13 columns (features). For clarification purposes, we slightly changed the names of some features of the original dataset (Additional file 1). We report the quantitative characteristics of the dataset in Table 2 and Table 3. Additional information about this dataset can be found in the original dataset curators publication [52, 66].

**Table 2:** Statistical quantitative description of the category features

	Full sample		Dead patients		Survived patients	
Category feature	#	%	#	%	#	%
Anaemia (0: false)	170	56.86	50	52.08	120	59.11
Anaemia (1: true)	129	43.14	46	47.92	3	40.89
High blood pressure (0: false)	194	64.88	57	59.38	137	67.49
High blood pressure (1: true)	105	35.12	39	40.62	66	32.51
Diabetes (0: false)	174	58.19	56	58.33	118	58.13
Diabetes (1: true)	125	41.81	40	41.67	85	41.87
Sex (0: woman)	105	35.12	34	35.42	71	34.98
Sex (1: man)	194	64.88	62	64.58	132	65.02
Smoking (0: false)	203	67.89	66	68.75	137	67.49
Smoking (1: true)	96	32.11	30	31.25	66	32.51

#: number of patients. %: percentage of patients. Full sample: 299 individuals. Dead patients: 96 individuals. Survived patients: 203 individuals.

**Table 3:** Statistical quantitative description of the numeric features

	Full sample			Dead patients			Survived patients		
Numeric feature	Median	Mean	*σ*	Median	Mean	*σ*	Median	Mean	*σ*
Age	60.00	60.83	11.89	65.00	65.22	13.21	60.00	58.76	10.64
Creatinine phosphokinase	250.00	581.80	970.29	259.00	670.20	1316.58	245.00	540.10	753.80
Ejection fraction	38.00	38.08	11.83	30.00	33.47	12.53	38.00	40.27	10.86
Platelets	262.00	263.36	97.80	258.50	256.38	98.53	263.00	266.66	97.53
Serum creatinine	1.10	1.39	1.03	1.30	1.84	1.47	1.00	1.19	0.65
Serum sodium	137.00	136.60	4.41	135.50	135.40	5.00	137.00	137.20	3.98
Time	115.00	130.30	77.61	44.50	70.89	62.38	172.00	158.30	67.74

Full sample: 299 individuals. Dead patients: 96 individuals. Survived patients: 203 individuals. *σ*: standard deviation

## Methods

In this section, we first list the machine learning methods we used for the binary classification of the survival (“Survival prediction classifiers” section), and the biostatistics and machine learning methods we employed for the feature ranking (“Feature ranking” section), discarding each patient’s follow-up time. We then describe the logistic regression algorithm we employed to predict survival and to perform the feature ranking as a function of the follow-up time (“Stratified logistic regression” section). We implemented all the methods with the open source R programming language, and made it publically freely available online (Data and software availability).

### Survival prediction classifiers

This part of our analysis focuses on the binary prediction of the survival of the patients in the follow-up period.

To predict patients survival, we employed ten different methods from different machine learning areas. The classifiers include one linear statistical method (Linear Regression [71]), three tree-based methods (Random Forests [72], One Rule [73], Decision Tree [74]), one Artificial Neural Network (perceptron [75]), two Support Vector Machines (linear, and with Gaussian radial kernel [76]), one instance-based learning model (*k*-Nearest Neighbors [77]), one probabilistic classifier (Naïve Bayes [78]), and an ensemble boosting method (Gradient Boosting [79]).

We measured the prediction results through common confusion matrix rates such as Matthews correlation coefficient (MCC) [80], receiver operating characteristic (ROC) area under the curve, and precision-recall (PR) area under the curve (Additional file 1) [81]. The MCC takes into account the dataset imbalance and generates a high score only if the predictor performed well both on the majority of negative data instances and on the majority of positive data instances [82–84]. Therefore, we give more importance to the MCC than to the other confusion matrix metrics, and rank the results based on the MCC.

### Feature ranking

For the feature ranking, we employed a traditional univariate biostatistics analysis followed by a machine learning analysis; afterwards, we compared the results of the two approaches.

**Biostatistics**. We used common univariate tests such as Mann–Whitney *U* test [85], Pearson correlation coefficient [86], and chi square test [87] to compare the distribution of each feature between the two groups (survived individuals and dead patients), plus the Shapiro–Wilk test [88] to check the distribution of each feature. Each test has a different meaning but all of them produce a score (a coefficient for the PCC, and a *p*-value for the other tests) representing the likelihood of a feature to be associated to the target. These scores can then be employed to produce a ranking, that lists the features from the most target-related to the least target-related.

The Mann–Whitney *U* test (or Wilcoxon rank–sum test) [85], applied to each feature in relation to the death event target, detects whether we can reject the null hypothesis that the distribution of the each feature for the groups of samples defined by death event are the same. A low *p*-value of this test (close to 0) means that the analyzed feature strongly relates to death event, while a high *p*-value (close to 1) means the opposite. The Pearson correlation coefficient (or Pearson product-moment correlation coefficient, PCC) [86] indicates the linear correlation between elements of two lists, showing the same elements on different positions. The absolute value of PCC generates a high value (close to 1) if the elements of the two lists have linear correlation, and a low value (close to 0) otherwise.

The chi square test (or *χ*^2^ test) [87] between two features checks how likely an observed distribution is due to chance [89]. A low *p*-value (close to 0) means that the two features have a strong relation; a high *p*-value (close to 1) means, instead, that the null hypothesis of independence cannot be discarded.

Similar to what Miguel and colleagues did on a breast cancer dataset [90], we decided also to take advantage of the Shapiro–Wilk test [88] to assess if each feature was extracted from a normal distribution.

**Machine learning**. Regarding machine learning feature ranking, we focused only on Random Forests [72, 91], because as it turned out to be the top performing classifier on the complete dataset (“Feature ranking results” section). Random Forests [72] provides two feature ranking techniques: mean accuracy reduction and Gini impurity reduction [92]. During training, Random Forests generates several random Decision Trees that it applies to data subsets, containing a subsets both of data instances and of features. In the end, Random Forests checks all the binary outcomes of these decisions trees and chooses its final outcome through a majority vote. The feature ranking based upon the mean accuracy decreases counts how much the prediction accuracy decreases, when a particular feature is removed. The method then compares this accuracy with the accuracy obtained by using all the features, and considers this difference as the *importance* of that specific feature: the larger the accuracy drop, the more important the feature. The other feature ranking method works similarly, but is based upon the Gini impurity decrease [91]: the more the Gini impurity drops, the more important the feature.

### Aggregate feature rankings and prediction on the top features

Starting from the whole dataset *D* we generated a collection $\mathcal {D}=\left \{\left \{D^{\text {tr}}_{i},D^{\text {ts}}_{i}\right \}\right \}_{i=1}^{N}$ of *N* Monte Carlo stratified training/test partitions $D=D^{\text {tr}}_{i} \cup D^{\text {ts}}_{i}$ with ratio 70%/30%.

For each execution, we randomly selected 70% of patients for the training set, and used the remaining 30% for the test set. To make our predictions more realistic, we avoided using the same balance ratio of the whole complete dataset (32.11% positives and 67.89% negatives). This way, we had different balance ratios for each of the 100 executions with, on average, 32.06% positives and 66.94% negatives on average in the training sets, and with, on average, 32.22% positives and 67.78% negatives on average in the test sets.

On the *N* training portions *D*1tr,…,*DN*tr we applied seven different feature ranking methods, namely RReliefF [93–95], Max-Min Parents and Children [96–98], Random Forest [72], One Rule [73], Recursive Partitioning and Regression Trees [99], Support Vector Machines with linear kernel [100] and eXtreme Gradient Boosting [79, 101, 102], using the feature death event as the target and obtaining 7*N* ranked lists of the 11 features. Agglomerating all the 7*N* features into the single Borda list [103, 104] we obtained the global list (Fig. 2 for *N*=100), together with the Borda count score of each feature, corresponding to the average position across all 7*N* lists, and thus the lower the score, the more important the feature.

We then used only the top–two features, namely serum creatinine and ejection fraction to build on each subset $D^{\text {tr}}_{i}$ three classifiers, namely Random Forests (RF), Support Vector Machine with Gaussian Kernel (GSVM) and eXtreme Gradient Boosting (XGB). Finally, we then applied the trained models to the corresponding test portions $D^{\text {ts}}_{i}$ with the aforementioned top–2 features and averaged the obtained performances modelwise on the *N* test set instances.

For the feature ranking and the classification made on the top two features, we employed different sets of the machine learning methods than the ones we used for the survival prediction on the complete dataset (“Survival prediction classifiers” section): RReliefF, Max-Min Parents and Children, Random Forests, One Rule, Recursive Partitioning and Regression Trees Support Vector Machines with linear kernel, and eXtreme Gradient Boosting, for the feature ranking, and Random Forests, Gradient Boosting, and SVM with radial kernel. We decided to use three different sets of methods because we aimed to demonstrate the generalisability of our approach, by showing that our computational solution is not only valid with few machine learning classifiers, but rather works for several groups of methods.

Regarding the final prediction using only the top two selected features, we chose Random Forests because it resulted in being the top performing classifier on the complete feature dataset (“Survival machine learning prediction on all clinical features” section) and it is universally considered an efficient method for feature ranking [92]. We then chose Gradient Boosting and Support Vector Machine with radial Gaussian kernel because both these methods have shown efficient performances in feature ranking with medical informatics data [105, 106].

### Stratified logistic regression

In the just-described first analysis, we wanted to predict the survival of patients and to detect the clinical feature importance in the follow-up time, without considering its different extent for each patient. In the second analysis, we decided to include the follow-up time, to see if the survival prediction results or the feature ranking results would change. To analyze this aspect, we mapped the original dataset time feature (containing the days of follow-up) into a month variable, where *month 0* means that fewer than 30 days have gone by, *month 1* means between 30 and 60 days, *month 2* means between 60 and 90 days, and so on.

We then applied a stratified logistic regression [107] to the complete dataset, including all the original clinical features and the derived follow-up month feature. We measured the prediction with the aforementioned confusion matrix metrics (MCC, F_1_ score, etc.), and the feature ranking importance as the logistic regression model coefficient for each variable.

## Results

In this section, we first describe the results we obtained for the survival prediction on the complete dataset (“Survival machine learning prediction on all clinical features” section), the results obtained for the feature ranking (“Feature ranking results” section), and the results on the survival prediction when using only the top two most important features of the dataset (“Survival machine learning prediction on serum creatinine and ejection fraction alone” section and “Serum creatinine and ejection fraction linear separability” section), all independently from the follow-up time. We then report and discuss the results achieved by including the follow-up time of each patient in the survival prediction and feature ranking (“Survival prediction and feature ranking including the follow-up period” section).

### Survival machine learning prediction on all clinical features

We employed several methods to predict the survival of the patients. We applied each method 100 times and reported the mean result score (Table 4).

**Table 4:** Survival prediction results on all clinical features – mean of 100 executions

Method	**MCC**	F_1_ score	Accuracy	TP rate	TN rate	PR AUC	ROC AUC
Random forests	blue+0.384*	0.547	blue0.740*	0.491	0.864	0.657	blue0.800*
Decision tree	**+0.376**	blue0.554*	0.737	blue0.532*	0.831	0.506	0.681
Gradient boosting	**+0.367**	0.527	0.738	0.477	0.860	0.594	0.754
Linear regression	**+0.332**	0.475	0.730	0.394	0.892	0.495	0.643
One rule	**+0.319**	0.465	0.729	0.383	0.892	0.482	0.637
Artificial neural network	**+0.262**	0.483	0.680	0.428	0.815	blue0.750*	0.559
Naïve bayes	**+0.224**	0.364	0.696	0.279	0.898	0.437	0.589
SVM radial	**+0.159**	0.182	0.690	0.122	0.967	0.587	0.749
SVM linear	**+0.107**	0.115	0.684	0.072	blue0.981*	0.594	0.754
*k*-nearest neighbors	**-0.025**	0.148	0.624	0.121	0.866	0.323	0.493

MCC: Matthews correlation coefficient. TP rate: true positive rate (sensitivity, recall). TN rate: true negative rate (specificify). Confusion matrix threshold for MCC, F_1_ score, accuracy, TP rate, TN rate: *τ*=0.5. PR: precision-recall curve. ROC: receiver operating characteristic curve. AUC: area under the curve. MCC: worst value = –1 and best value = +1. F_1_ score, accuracy, TP rate, TN rate, PR AUC, ROC AUC: worst value = 0 and best value = 1. MCC, F_1_ score, accuracy, TP rate, TN rate, PR AUC, ROC AUC formulas: Additional file 1 (“Binary statistical rates” section). Gradient boosting: eXtreme Gradient Boosting (XGBoost). SVM radial: Support Vector Machine with radial Gaussian kernel. SVM linear: Support Vector Machine with linear kernel. Our hyper-parameter grid search optimization for *k*-Nearest Neighbors selected *k*=3 on most of the times (10 runs out of 100). Our hyper-parameter grid search optimization for the Support Vector Machine with radial Gaussian kernel selected *C*=10 on most of the times (56 runs out of 100). Our hyper-parameter grid search optimization for the Support Vector Machine with linear kernel selected *C*=0.1 on most of the times (50 runs out of 100). Our hyper-parameter grid search optimization for the Artificial Neural Network selected 1 hidden layer and 100 hidden units on most of the times (74 runs out of 100). We report bluein blue and with ^∗^ the top performer results for each score.

For methods that needed hyper-parameter optimization (neural network, Support Vector Machine, and *k*-Nearest Neighbors), we split the dataset into 60% (179 randomly selected patients) for the training set, 20% (60 randomly selected patients) for the validation set, and 20% (the remaining 60 patients) for the test set. To choose the top hyper-parameters, we used a grid search and selected the models that generated the highest Matthews correlation coefficient [83].

For the other methods (Random Forests, One Rule, Linear Regression, Naïve Bayes, and Decision Tree), instead, we split the dataset into 80% (239 randomly selected patients) for the training set, and 20% (the remaining 60 patients) for the test set.

For each of the 100 executions, our script randomly selected data instances for the training set and for the test (and for the validation set, in the case of hyper-parameter optimization) from the complete original dataset. We trained the model on the training set (and validated it on the validation set, in the case of hyper-parameter optimization). We then applied the script to the test set. Given the different selections of data instances for the dataset splits, each execution led to slightly different results.

Our prediction results showed that Random Forests outperformed all the other methods, by obtaining the top MCC (+0.384), the top accuracy (0.740), and the top ROC AUC (0.800) (Table 4). The Decision Trees obtained the top results on the true positives (sensitivity = 0.532) and on the F_1_ score (0.554), and was the only classifier able to predict correctly the majority of deceased patients. The linear Support Vector Machines achieved an almost perfect prediction score on the negative elements (specificity = 0.961), but a poor score on the positive elements (sensitivity = 0.072). The Artificial Neural Network perceptron, instead, obtained the top value on the Precision-Recall AUC (0.750).

Because of the imbalance of the dataset (67.89% negative elements and 32.11% positive elements), all the methods obtained better prediction scores on the true negative rate, rather than on the true positive rate (Table 4). These results occur because the algorithms can see more negative elements during training, and therefore they are more trained to recognize deceased patient profiles during testing.

### Feature ranking results

Similarly to what authors did for a dataset of patients having mesothelioma symptoms [92], we decided then to investigate the most important features of the cardiovascular heart disease patients dataset. To this aim, we first performed a traditional univariate biostatistics analysis (“Feature ranking” section), and then employed Random Forests [108], to generate machine learning results. We then compared the results obtained through the two approaches.

All the biostatistics tests (Mann–Whitney *U* test in Table 5, Pearson correlation coefficient in Table 6, and chi squared test in Table 7) identified serum creatinine and ejection fraction as the top two most important features.

**Table 5:** Mann–Whitney *U* test

		Mann–Whitney *U*
Rank	Feature	Test *p*-value
1	Serum creatinine	0
2	Ejection fraction	0.000001
3	Age	0.000167
4	Serum sodium	0.000293
5	High blood pressure	0.171016
6	Anaemia	0.252970
7	Platelets	0.425559
8	Creatinine phosphokinase	0.684040
9	Smoking	0.828190
10	Sex	0.941292
11	Diabetes	0.973913

Results of the univariate application of the Mann–Whitney *U* test between each feature and the target feature death event

**Table 6:** Pearson correlation coefficients (PCC) and Shapiro–Wilk tests

Pearson correlation coefficient			Shapiro–Wilk test		
Rank	Feature	abs(PCC)	Rank	Feature	*p*-value
1	Serum creatinine	0.294	1	Creatinine phosphokinase	7.05×10^−28^
2	Ejection fraction	0.269	2	Serum creatinine	5.39×10^−27^
3	Age	0.254	3	Smoking	4.58×10^−26^
4	Serum sodium	0.195	4	Death event	4.58×10^−26^
5	High blood pressure	0.079	5	Sex	1.17×10^−25^
6	Anaemia	0.066	6	High blood pressure	1.17×10^−25^
7	Creatinine phosphokinase	0.063	7	Diabetes	5.12×10^−25^
8	Platelets	0.049	8	Anaemia	6.21×10^−25^
9	Smoking	0.013	9	Platelets	2.89×10^−12^
10	Sex	0.004	10	Serum sodium	9.21×10^−10^
11	Diabetes	0.002	11	Ejection fraction	7.22×10^−09^
			12	Age	5.34×10^−05^

Results of the univariate application of the Pearson correlation coefficient between each feature and the target feature death event, absolute value (left), and the univariate application of the Shapiro–Wilk test on each feature (right)

**Table 7:** Chi squared test

Chi squared test		
Rank	Feature	*p*-value
1	Ejection fraction	0.000500
2	Serum creatinine	0.000500
3	Serum sodium	0.003998
4	Age	0.005997
5	High blood pressure	0.181909
6	Anaemia	0.260370
7	Creatinine phosphokinase	0.377811
8	Platelets	0.637681
9	Smoking	0.889555
10	Sex	1
11	Diabetes	1

Results of the application of the chi squared test between each feature and the target feature death event

Mann–Whitney *U* test (Table 5) and chi squared test (Table 7), in particular, showed a significant *p*-value close to 0 for both these two features. The Pearson correlation coefficient results (Table 6, left side) also showed age, in the third position, as a top feature among serum creatinine and ejection fraction.

The Shapiro–Wilk test (Table 6, right side) generated *p*-values close to 0 for all the features, meaning that the null hypothesis of normality is rejected, and all variables are non-normal.

**Fig. 1:**
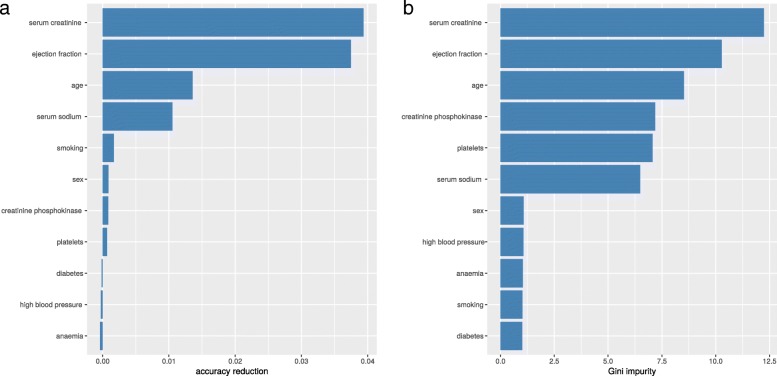
Random Forests feature selection. Accuracy reduction. Gini impurity. Random Forests feature selection through accuracy reduction (**a**). Random Forests feature selection through Gini impurity (**b**)

Regarding Random Forests feature ranking, both the accuracy reduction and the Gini impurity rankings detected serum creatinine, ejection fraction, and age as the top three most important features of the dataset (Fig. 1). The two rankings show high similarity: the Kendall *τ* rank correlation coefficient between them is +0.56 and the Spearman *ρ* rank correlation coefficient is +0.73. Both these coefficients range between −1 (when the ranking of a list is the opposite of the other one) and +1 (when the two rankings are similar) [109].

**Fig. 2:**
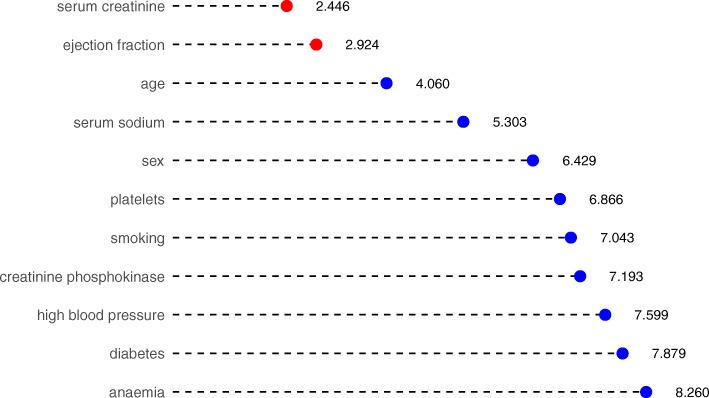
Aggregated results of the feature rankings. Borda list of the 700 rankings obtained applying seven ranking methods on 100 instances of 70% training subsets of *D*. We ranked the Borda list by importance, quantitatively expressed as the Borda count score, corresponding to the average position across all 700 lists. The lower the score, the higher the average rank of the feature in the 700 lists and thus the more important the feature. We highlight the top two features with red circles

To have a unique final classification to evaluate, we then merged the two rankings into an aggregate ranking by using Borda’s method [110]. For every feature *f*, we added its position in the accuracy decrease ranking *p*_1_(*f*) to its position in the Gini impurity raking *p*_2_(*f*), and saved this aggregate value in the ranking variable *score*_*f*_. Finally, we sorted all the features increasingly based upon *score*_*f*_ (Table 8).

**Table 8:** Random Forests feature selection aggregate ranking

Final rank	Feature	Accuracy decrease	Accuracy decrease rank	Gini impurity	Gini impurity rank
1	Serum creatinine	3.78×10^−2^	1	11.84	1
2	Ejection fraction	3.43×10^−2^	2	10.71	2
3	Age	1.53×10^−2^	3	8.58	3
4	Creatinine phosphokinase	7.27×10^−4^	6	7.26	4
4	Serum sodium	7.20×10^−3^	4	6.49	6
6	Sex	1.64×10^−3^	5	1.12	8
6	Platelets	2.47×10^−4^	8	6.80	5
8	High blood pressure	−1.68×10^−3^	11	1.13	7
8	Smoking	3.68×10^−4^	7	0.95	11
10	Anaemia	−5.91×10^−4^	10	1.06	9
10	Diabetes	−1.41×10^−4^	9	1.02	10

We merged the two rankings through their position, through the Borda’s method [103]

In the aggregated ranking (Table 8), creatinine phosphokinase appeared as the fourth most important feature tied with serum sodium, while anaemia and diabetes were the least important features among all.

Once we obtained the ranking of the features based upon their importance, we aimed to understand what is the minimum number of features (and which features should be used) to still be able to perform an accurate prediction of the survival of patients. In fact, we want to provide a method that can be used by medical doctors in the hospital, in the scenario where just few features of the electronic health record (EHR) of a patient are available.

Since we observed that serum creatinine and ejection fraction resulted as the top two features in the univariate biostastitics analysis tests (Pearson correlation coefficient in Table 6, Mann–Whitney *U* test in Table 5 and chi squared in Table 7), and in the Random Forests analysis (Table 8 and Fig 1), we decided to explore if it is possible to correctly predict the patients’ survival from these top two clinical features alone.

### Survival machine learning prediction on serum creatinine and ejection fraction alone

As mentioned earlier (“Aggregate feature rankings and prediction on the top features” section), we decided to investigate if machine learning can precisely predict patients’ survival by using the top two ranked features alone. We therefore elaborated another computational pipeline with an initial phase of feature ranking, followed by a binary classification phase based on the top two features selected (Table 9).

**Table 9:** Survival prediction results on serum creatinine and ejection fraction – mean of 100 executions

Method	**MCC**	F_1_ score	Accuracy	TP rate	TN rate	PR AUC	ROC AUC
Random forests	blue+0.418*	blue0.754*	blue0.585*	0.541	blue0.855*	0.541	0.698
Gradient boosting	**+0.414**	0.750	blue0.585*	blue0.550*	0.845	blue0.673*	blue0.792*
SVM radial	**+0.348**	0.720	0.543	0.519	0.816	0.494	0.667

MCC: Matthews correlation coefficient. TP rate: true positive rate (sensitivity, recall). TN rate: true negative rate (specificify). Confusion matrix threshold for MCC, F_1_ score, accuracy, TP rate, TN rate: *τ*=0.5. PR: precision-recall curve. ROC: receiver operating characteristic curve. AUC: area under the curve. MCC: worst value = –1 and best value = +1. F_1_ score, accuracy, TP rate, TN rate, PR AUC, ROC AUC: worst value = 0 and best value = 1. MCC, F_1_ score, accuracy, TP rate, TN rate, PR AUC, ROC AUC formulas: Additional file 1 (“Binary statistical rates” section). Gradient boosting: eXtreme Gradient Boosting (XGBoost). SVM radial: Support Vector Machine with radial Gaussian kernel. We reported bluein blue and with ^∗^ the top results for each score.

All the different methods employed for feature ranking identified serum creatinine and ejection fraction as the top two features for all the 100 executions (Fig. 2), so we then performed a survival prediction on these two features by employing three algorithms: Random Forests, Gradient Boosting, and SVM radial.

All the three classifiers employed outperformed their corresponding results obtained on the complete datase (Table 4). Random Forests and Gradient Boosting applied to serum creatinine and ejection fraction alone, moreover, even outperformed all the methods applied to the complete dataset (Table 4), by achieving Matthews correlation coefficients greater than +0.4 in the [−1; +1] range. Similar to the application on the complete dataset, here the classifiers obtained better results on the specificity (true negative rate) than on the recall (true positive rate), because of the imbalance of the dataset.

### Serum creatinine and ejection fraction linear separability

**Fig. 3:**
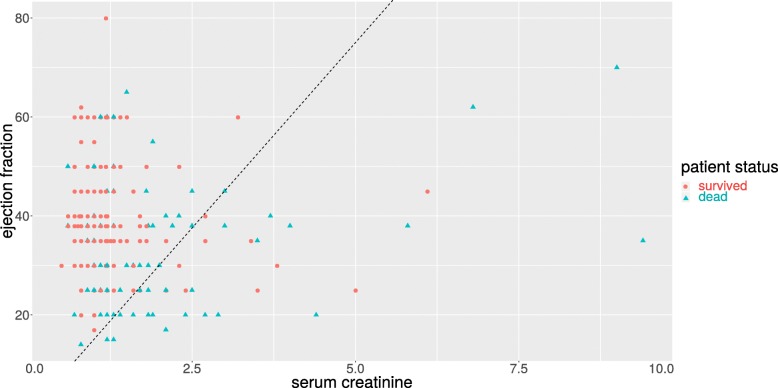
Scatterplot of serum creatinine versus ejection fraction. Serum creatinine (x axis) range: [0.50, 9.40] mg/dL. Ejection fraction (y axis) range: [14, 80]%. We manually drew a black straight line to highlight the discrimination between alive and dead patients

To verify further the predictive power of serum creatinine and ejection fraction, we depicted a scatterplot with the serum creatinine values on the *x* axis and the ejection fraction values on the *y* axis, and we colored every patient-point based on survival status (survived or dead, Fig. 3). This plot shows a clear distinction between alive patients and dead patients, that we highlighted by manually inserting a black straight line.

### Survival prediction and feature ranking including the follow-up period

In the previous part of the analysis, we excluded follow-up time from the dataset because we prefered to focus on the clinical features and to try to discover something meaningful about them. Follow-up time, however, can be an important factor in the survival of patients, and should not be eliminated completely from this study. We therefore decided to investigate the possible relationship between follow-up time and the survival of patients: is the moment of the follow-up visit related to the chance of survival of the patient?

**Fig. 4:**
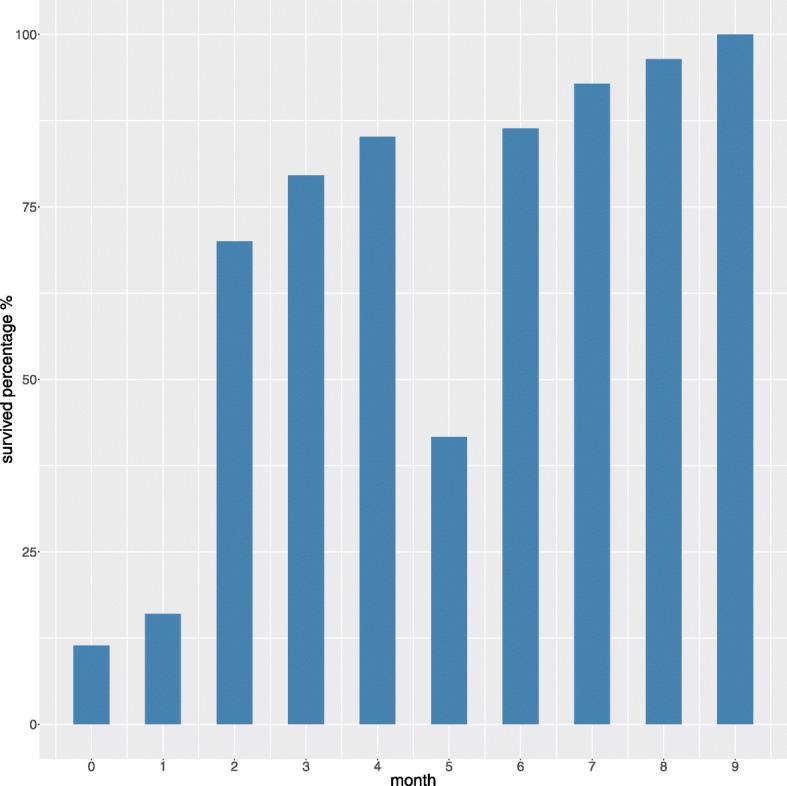
Barplot of the survival percentage for each follow-up month. Follow-up time (x axis) range: [0, 9] months. Survival percentage (y axis) range: [11.43, 100]%. For each month, we report here the percentage of survived patients. For the 0 month (less than 30 days), for example, there were 11.43% survied patients and 88.57% deceased patients

**Follow-up time and survival**. To analyze this aspect, we first grouped together all the surviving patients and the deceased patients for each month. We then built a barplot that relates the percentage of surviving patients to each follow-up month (Fig. 4). This plot shows that it is impossible to correlate the survival of patients to the follow-up month because the survival trend is not linear: the *month 5*, in fact, reports less surviving patients than *month 4* and *month 6* (Fig. 4).

For the same reasons, there is no trend showing an increasing or decreasing rate of survived patients as function of months gone by: the *month 5*, in fact, has less half survived patients, similarly to *month 0* and *month 1*, without being adjacent to them (Fig. 4).

**Survival prediction including follow-up time**. Even if we notice no linear correlation between follow-up month and survival, we decided to repeat the survival prediction analysis and the feature ranking analysis by including this feature, and to explore the relevance of ejection fraction and serum creatinine in this case. As mentioned earlier (“Stratified logistic regression” section), we used a stratified logistic regression for this task.

We first applied the logistic regression to rank all the clinical features. The results we obtained (Table 10) again showed ejection fraction and serum creatinine to be the most relevant clinical factors.

**Table 10:** Stratified logistic regression feature ranking

Rank	Clinical feature	Importance
1	Ejection fraction	4.13938106
2	Serum creatinine	3.69917184
3	Age	2.61938095
4	Creatinine phosphokinase	1.88929235
5	Sex	1.32038950
6	Platelets	1.06270364
7	High blood pressure	0.79478093
8	Anaemia	0.77547306
9	Smoking	0.65828165
10	Diabetes	0.60355319
11	Serum sodium	0.54241360

Results of the feature ranking obtained by the stratified logisitc regression. Importance: coefficient of the trained logistic regression model, average of 100 execution

**Table 11:** Survival prediction results including the follow-up time – mean of 100 executions

Method	**MCC**	F_1_ score	Accuracy	TP rate	TN rate	PR AUC	ROC AUC
Logistic regression	blue+0.616*	blue0.719*	blue0.838*	blue0.785*	blue0.860*	blue0.617*	blue0.822*
(EF, SR, & FU)							
Logistic regression	**+0.607**	0.714	0.833	0.780	0.856	0.612	0.818
(all features)							

Top row: logistic regression using only ejection fraction (EF), serum creatinine (SC), and follow-up time month (FU). Bottom row: logistic regression using all features. MCC: Matthews correlation coefficient. TP rate: true positive rate (sensitivity, recall). TN rate: true negative rate (specificify). Confusion matrix threshold for MCC, F_1_ score, accuracy, TP rate, TN rate: *τ*=0.5. PR: precision-recall curve. ROC: receiver operating characteristic curve. AUC: area under the curve. MCC: worst value = –1 and best value = +1. F_1_ score, accuracy, TP rate, TN rate, PR AUC, ROC AUC: worst value = 0 and best value = 1. MCC, F_1_ score, accuracy, TP rate, TN rate, PR AUC, ROC AUC formulas: Additional file 1 (“Binary statistical rates” section). We reported bluein blue and with ^∗^ the top results for each score.

We trained the model on the whole dataset, and then ranked the non-temporal features based upon their generalized linear model (GLM) coefficients. We repeated this operation 100 times and reported the average importance value for each variable (Table 10).

Afterwards, in a similar way as we did before (“Aggregate feature rankings and prediction on the top features” section), we decided to investigate how the survival prediction would behave when using only the two selected features. We trained the stratified logistic regression on all the features including the follow-up time, by using 70% of patients, randomly selected. We then selected the top two clinical features, trained a model by using only these top two features and follow-up month, and tested this three-feature model on the test set. We applied this method 100 times, and reported the average results (Table 11). For all executions, the top two features were ejection fraction and serum creatinine.

The stratified logistic regression using only three features outperformed the model using all features, in each confusion matrix. metric (Table 10). The results showed that, when including follow-up month into the model, machine learning predictions using only ejection fraction and serum creatinine outperformed a prediction using all the clinical features.

Additionally, the results obtained by the stratified logistic regression and considering the follow-up month (Table 10) outperformed the results achieved by the other methods without the follow-up month (Table 4 and Table 9), highlighting the importance of this temporal variable.

## Discussion

Our results not only show that B it might be possible to predict the survival of patients with heart failure solely from their serum creatinine and ejection fraction, but also that the prediction made on these two features alone can be more accurate than the predictions made on the complete dataset. This aspect is particularly encouraging for the hospital settings: in case many laboratory test results and clinical features were missing from the electronic health record of a patient, doctors could still B be able to predict patient survival by just analyzing the ejection fraction and serum creatinine values. B That being said, we recognize that additional confirmatory studies need to be completed before this machine learning procedure can be taken up into clinical practice.

Our analysis also generated some interesting results that differ from the original dataset curators study [52]. Ahmad and colleagues, in fact, identified age, serum creatinine (renal dysfunction), high blood pressure, ejection fraction and anaemia as top features. In our Random Forests feature ranking instead (Table 8), high blood pressure is on 8^*th*^ position out of 11, and anaemia is on the 10^*th*^ position out of 11 (last position tied with diabetes).

## Conclusions

In our work, the fact that our traditional biostatistics analysis selected ejection fraction and serum creatinine as the two most relevant features confirmed the relevance of the feature ranking executed with machine learning. Moreover, our approach showed that machine learning can be used effectively for binary classification of electronic health records of patients with cardiovascular hearth diseases.

As a limitation of the present study, we have to report the small size of the dataset (299 patients): a larger dataset would have permitted us to obtain more reliable results. Additional information about the physical features of the patients (height, weight, body mass index, etc.) and their occupational history would have been useful to detect additional risk factors for cardiovascular health diseases. Also, if an additional external dataset with the same features from a different geographical region had been available, we would have used it as a validation cohort to verify our findings.

Regarding future developments, we plan to apply our machine learning approach to alternative datasets of cardiovascular heart diseases [111–113] and other illnesses (cervical cancer [114], neuroblastoma [115], breast cancer [90], and amyotrophic lateral sclerosis [116]).

## Supplementary information

**Additional file 1** Supplementary information.

## Data Availability

The dataset used in this project [66] is publicly available under the Creative Commons Attribution 4.0 International (CC BY 4.0) license at: https://plos.figshare.com/articles/Survival_analysis_of_heart_failure_patients_A_case_study/5227684/1 Our software code is publicly available under the GNU General Public License v3.0 at: https://github.com/davidechicco/cardiovascular_heart_disease

## Abbreviations

AUC: area under the curve
CPK: creatinine phosphokinase
CVDs: cardiovascular diseases
EF: ejection fraction
EHR: electronic health records
EU: European Union
FU: follow-up
GLM: generalized linear model
GSVM: Support Vector Machine with Gaussian Kernel
HF: heart failure
HFpEF: heart failure with preserved ejection fraction
HFrEF: heart failure due to reduced ejection fraction
LV: left ventricular
MCC: Matthews correlation coefficient
NYHA: New York Heart Association
PCC: Pearson correlation coefficient
PR: precision-recall
RF: Random Forests
ROC: receiver operating characteristic
SC: serum creatinine
SNPs: single-nucleotide polymorphisms
SVM: Support Vector Machine
TN rate: true negative rate
TP rate: true positive rate
XGB: eXtreme Gradient Boosting
