# Supplementary material for: Machine learning can predict survival of patients with heart failure from serum creatinine and ejection fraction alone
Source: BMC Med Inform Decis Mak. 2020 Feb 3;20:16. doi: 10.1186/s12911-020-1023-5 (PMC6998201; doi:10.1186/s12911-020-1023-5)
Supplement: Supplementary file 1 — Additional file 1 Supplementary information. [file 12911_2020_1023_MOESM1_ESM.pdf]

Supplementary information

Original feature renaming

For clarification and precision purposes, we slightly changed some names of the features from the original dataset [?, ?]:
We renamed ”Gender” to ”sex”.
We renamed ”BP” to ”high blood pressure”.
We renamed ”Platelets” to ”platelets”.
We renamed ”Sodium” to ”serum sodium”.
We renamed ”CPK” to ”creatinine phosphokinase”.
We renamed ”Creatinine” to ”serum creatinine”.
We renamed ”Event” to ”death event”, which is the survival target.

Binary statistical rates

List of statistical rates and their formulas:

$$\text{MCC} = \frac{TP \cdot TN - FP \cdot FN}{\sqrt{(TP+FP)\cdot(TP+FN)\cdot(TN+FP)\cdot(TN+FN)}} \tag{1}$$

(worst value = −1; best value = +1)

$$\text{F}_1 \text{ score} = \frac{2 \cdot TP}{2 \cdot TP + FP + FN} \tag{2}$$

(worst value = 0; best value = 1)

$$\text{accuracy} = \frac{TP + TN}{TP + FN + TN + FP} \tag{3}$$

(worst value = 0; best value = 1)

$$\text{true positive rate} = \text{recall} = \text{sensitivity} = \frac{TP}{TP + FN} \tag{4}$$

(worst value = 0; best value = 1)

$$\text{true negative rate} = \text{specificity} = \frac{TN}{TN + FP} \tag{5}$$

(worst value = 0; best value = 1)

$$\text{precision} = \frac{TP}{TP + FP} \tag{6}$$

(worst value = 0; best value = 1)

$$\text{false positive rate} = \text{fallout} = \frac{FP}{FP + TN} \tag{7}$$

(worst value = 1; best value = 0)

$$\text{Precision-Recall (PR) curve} = \begin{cases} true\ positive\ rate & on\ the\ x\ axis \\ precision & on\ the\ y\ axis \end{cases} \tag{8}$$

(worst value = 0; best value = 1)

$$\text{ROC curve} = \begin{cases} false\ positive\ rate & on\ the\ x\ axis \\ true\ positive\ rate & on\ the\ y\ axis \end{cases} \tag{9}$$

(worst value = 0; best value = 1)
